# Prognostic Implications of Antibodies to Soluble Liver Antigen in Autoimmune Hepatitis: A PRISMA-Compliant Meta-Analysis

**DOI:** 10.1097/MD.0000000000000953

**Published:** 2015-06-12

**Authors:** Zhi-Xian Chen, Jian-Guo Shao, Yi Shen, Jian Zhang, Yu Hua, Lu-Jun Wang, Gang Qin

**Affiliations:** From the Department of Clinical Pharmacy, Nantong Health College of Jiangsu Province, China (Z-XC); Center for Liver Diseases, Nantong Third People's Hospital, Nantong University, China (J-GS, YH, L-JW, GQ); and Department of Epidemiology and Medical Statistics, Nantong University, China (YS, JZ).

## Abstract

Prognostic evaluation is important for the management of patients with autoimmune hepatitis (AIH). Although some autoantibodies have been associated with disease activity and outcomes, the implication of antibodies to soluble liver antigen (anti-SLA) remains controversial.

To conduct a meta-analysis of observational studies which addressed differences in clinical characteristics by anti-SLA status in patients with AIH.

Three databases PUBMED, EMBASE, and OVID were systemically searched up to January 2015 using the terms “soluble liver antigen” or “liver-pancreas antigen” and “autoimmune hepatitis” with restriction to English-language.

Studies were included if at least 50 patients with objective diagnosis of AIH were enrolled, anti-SLA detection was performed for the patients, and prognostic outcomes and/or disease severity were reported.

Two investigators independently reviewed retrieved literature and evaluated eligibility. Discrepancy was resolved by discussion and a third investigator. Quality of included studies was evaluated using Newcastle-Ottawa Quality Assessment Scale (NOS). Data were pooled using fixed-effect or random-effect models.

Prognostic outcomes included death from hepatic failure or requirement for liver transplantation, and responses to immunosuppressive therapy regarding remission or relapse. Results were combined on the odds ratio (OR) or standardized mean difference (SMD) scales.

Eight studies were enrolled in this study, involving a total of 1297 AIH patients among whom 195 with anti-SLA. Pooled serum AST levels tended to be lower in anti-SLA seropositive patients. The presence of anti-SLA conferred 3.1-fold increased risk of hepatic death in AIH patients. The remission rates were comparable between anti-SLA seropositive and seronegative AIH patients, while anti-SLA positivity was associated with nearly 2-fold increased risk of relapse after drug withdrawal. Human leukocyte antigen (HLA) allotype DR3 was positively associated with anti-SLA.

Antibodies to SLA may be an indicator of increased risks of hepatic death and treatment relapse for AIH patients. Our findings suggest that the anti-SLA seropositive patients should be maintained indefinitely on individually adjusted medication to improve their prognosis.

## Abstract 2

Supplemental Digital Content is available in the text

## INTRODUCTION

The epidemiological data on autoimmune hepatitis (AIH) in the United States as a whole are still lacking. According to data collected between 1984 and 2000, the prevalence of AIH in the Alaska native population is as high as 42.9 per 100,000 population.^1^ In Europe, the incidence rates were 1.68 and 0.85 per 100,000 population per year in Demark and Sweden, respectively.^2,3^ In Asia, there are no robust epidemiological data on AIH. AIH has become a relatively common autoimmune liver disease.

The disease presentation of AIH ranges from asymptomatic to symptomatic onset, acute, chronic, or fulminant. The key consequences of AIH are cirrhosis, liver failure, and hepatocellular carcinoma (HCC), leading to death or requirement of liver transplantation (LT). Although asymptomatic patients account for nearly one third of total patients,^4^ AIH is a “not-so-silent” disease. Histological findings, including the frequency of cirrhosis, are similar between asymptomatic patients and symptomatic patients.^5^ Before the widespread use of immunosuppressive agents for AIH, as many as 40% of patients with untreated severe disease died within 6 months of diagnosis.^6^

Antibodies against soluble liver antigen (SLA) were first described in 1987 in a specific form of AIH.^7^ Antibodies to SLA and liver-pancreas antigen were originally defined as different antibodies but subsequently have been identified as the same autoantibody given that they share the same antigenic target.^8^ Anti-SLA antibodies can be detected by radioimmunoassay, enzyme-linked immunosorbent assays, immunoblotting, or dotblotting assays, rather than by immunofluorescence. The native cytosolic antigen or recombinant 50 kDa protein identified as *O*-phosphoseryl-tRNA: selenocysteinyl-tRNA synthase used in detection assays was confirmed by mass spectrometry with human native antigen in 2010.^9,10^ It has been suggested that anti-SLA antibody might be a marker of disease severity in patients with AIH.^11^ Several studies demonstrated that anti-SLA was associated with relapse after corticosteroid therapy.^12,13^ However, a recent report indicated that anti-SLA may be not associated with treatment response and outcome.^14^

The prognostic value of anti-SLA has been addressed by a few studies but still remain controversial. We conduct this meta-analysis to determine whether the anti-SLA seropositivity could define a distinct subset of AIH patients, in terms of clinical characteristics, treatment responses, and prognostic outcomes.

## METHODS

### Data Sources

Preferred reporting items for systematic review and meta-analysis (PRISMA) protocols were followed for of the conduct of the current study.^15^ PUBMED, EMBASE, and OVID database were searched up to January 2015. The search strategy included in terms of “antibody to soluble liver antigen,” “anti-SLA,” “antibody to liver-pancreas” or “anti-liver-pancreas antigen,” and “autoimmune hepatitis.” No limits were appointed in country, race, or publication year. In order to identify more relevant studies, we scanned and hand-searched references of retrieved articles and conference proceedings.

### Study Selection

The initial study selection was performed by review of titles and abstracts. If the articles seemed possibly relevant, the full texts were downloaded and reviewed for data retrieval.

To be included in this analysis, the studies should met the following criteria: types of studies—observational studies including cohort, cross-sectional, and case-control studies; types of participants—patients with objective diagnosis of AIH, and anti-SLA detection was performed for them; disease severity markers—laboratory tests such as aspartate aminotransferase (AST), total bilirubin (TBIL) immunoglobulin G (IgG) levels, evidence of cirrhosis, or histological examination; at least 1 of the following prognostic outcomes—death from liver failure or LT, responses to immunosuppressive therapy such as remission and relapse; a minimum sample size of 50; and published in English.

Study eligibility was assessed by 2 investigators independently. Discrepancy was resolved by discussion and consultation with a third investigator.

### Data Extraction

The collected information about study characteristics included: country, publication year, study design, number of patients, gender distribution, mean age, criteria for diagnosis of AIH, anti-SLA status, length of follow-up, laboratory and histological findings, prognosis such as hepatic death or LT, or responses to immunosuppressive therapy such as remission and relapse.

Newcastle-Ottawa quality assessment scale (NOS) was used to evaluate the quality of the enrolled studies, as we described elsewhere.^16^ Briefly, when a study had relevant information that could be related with NOS criteria, 1 point was added. A total of 8 items in cohort or case–control studies and 5 items in cross-sectional studies that could be associated with NOS were identified.

Two investigators independently extracted data and judged the quality of studies. Discrepancy was resolved by discussion and a third investigator.

### Statistical Analysis

Dichotomous variables were presented as odds ratios (ORs) with 95% confidence intervals (CIs), while continuous variables as standardized mean differences (SMDs) with 95% CIs. All measures of dispersion were described as standard deviations (SDs). Sometimes estimated SDs were derived from standard errors (SEs) from the studies. Statistical heterogeneity was determined by χ^2^ and inconsistency (I^2^) statistics, an I^2^ value greater than 50% represented heterogeneity.^16^ A fixed-effect model was applied for meta-analysis if there was no heterogeneity; otherwise, a random-effect model was adopted. Overall effects were evaluated using the Z test. Publication bias was assessed visually by funnel plots, and statistically by a rank correlation test (Begg test) and a regression asymmetry test (Egger test).

The meta-analysis was performed with Stata software version 12.0 (Stata Corp, TX), with significance set at *P* < 0.05.

## RESULTS

### Literature Search

**FIGURE 1:**
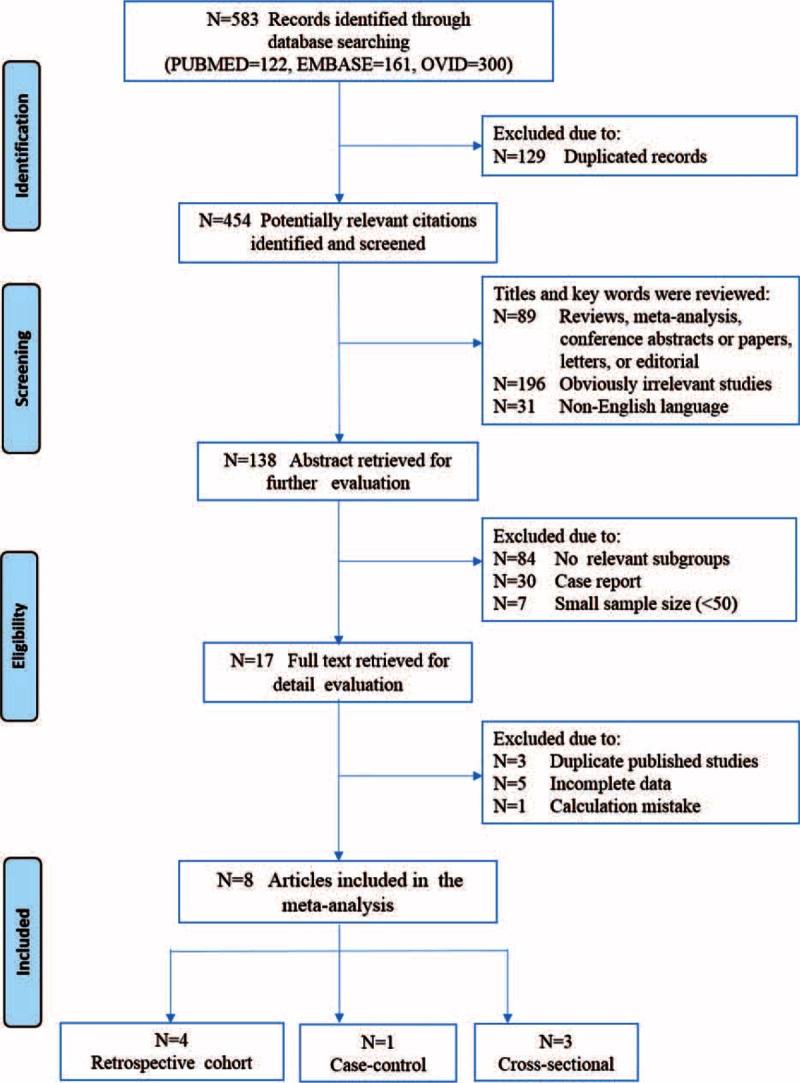
Flowchart of study selection for the meta-analysis.

The search procedures are summarized in Figure 1 with details. Briefly, we identified a total of 454 potentially relevant articles through online database search. By reviewing the titles and abstracts, 316 and 101 studies were excluded, respectively. Among the 37 full-text articles retrieved, 29 studies were excluded for lack of relevant subpopulations, small sample size, duplicate publications, or missing data. Eventually, 8 articles, published between 1999 and 2015, were eligible for enrollment in the present meta-analysis.

### Study Characteristics

Among the 8 enrolled studies, 4 were retrospective cohorts,^17–20^ 3 were cross-sectional,^12,13,21^ and 1 was case–control study.^14^ Two studies were multi-center studies.^13,18^ A total of 1297 AIH patients, among whom 195 were anti-SLA seropositive, were involved.

Countries involved in these studies were USA, Germany, France, Italy, Turkey, Japan, and Greece. All of the 8 included studies used the similar diagnostic criteria of AIH proposed by International Autoimmune Hepatitis Group in 1993, 1999, or 2008. A total of 7 studies included corticosteroid monotherapy or combination therapy consisting of azathioprine and corticosteroid, only 1 study used mycophenolate mofetil as first line therapy.^14^ The length of follow-up ranged from 1 to 407 months. The main characteristics of the selected studies are reported in Tables 1 and 2.

**TABLE 1:**
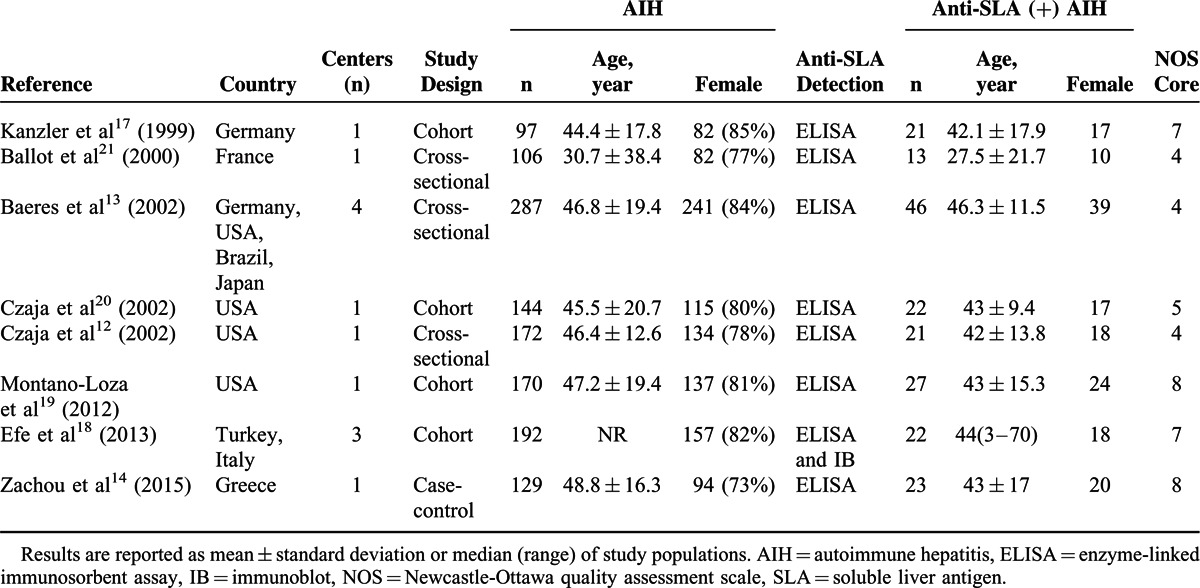
Summary Characteristics of the Selected Studies

**TABLE 2:**
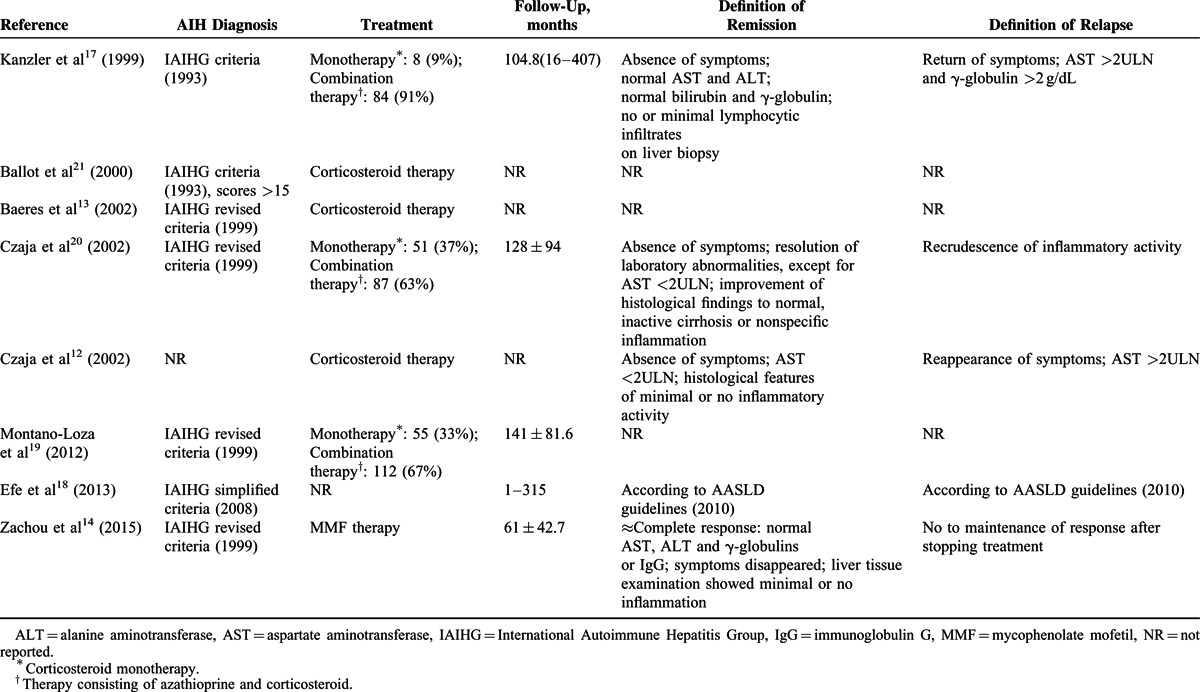
Diagnosis and Treatment of AIH in the Selected Studies

### Association of Anti-SLA with Disease Severity of AIH

Serum biochemical parameters were assessed in all studies. However, AST and TBIL levels with mean and SD values for anti-SLA positive and negative subgroups were available in 4 studies, while IgG was available in only 3 studies. Liver biopsy was conducted in 3 studies. Histologic inflammation scores were reported in 2 studies, while fibrosis index only in 1 study.

Data regarding AST extracted came from 583 AIH patients (anti-SLA positive in 91 cases and negative in 492 cases), and the analysis showed that the pooled mean AST levels were significantly lower in seropositive patients than in seronegative patients, according to the fixed-effect model (standardized mean difference −0.289, 95% CI −0.514 to −0.064, *P* = 0.012). Analysis about TBIL data which came from the same subjects showed that the summary mean bilirubin levels were comparable between the 2 groups of patients (*P* > 0.05). One study was excluded from the analysis of IgG because the authors did not disclose relevant data, the analysis showed that the standardized difference in mean values of IgG levels was not significantly different in anti-SLA positive AIH patients from negative ones.

In addition, 2 homogeneous reports involving 189 AIH patients (anti-SLA positive in 30 cases and negative in 159 cases) reported data about liver histological scores for inflammation. There was no significant association between anti-SLA and the inflammation scores in the fixed-effect model.

One study with histological data of fibrosis index showed that anti-SLA was not associated with the degree of liver fibrosis (Table 3).

**TABLE 3:**
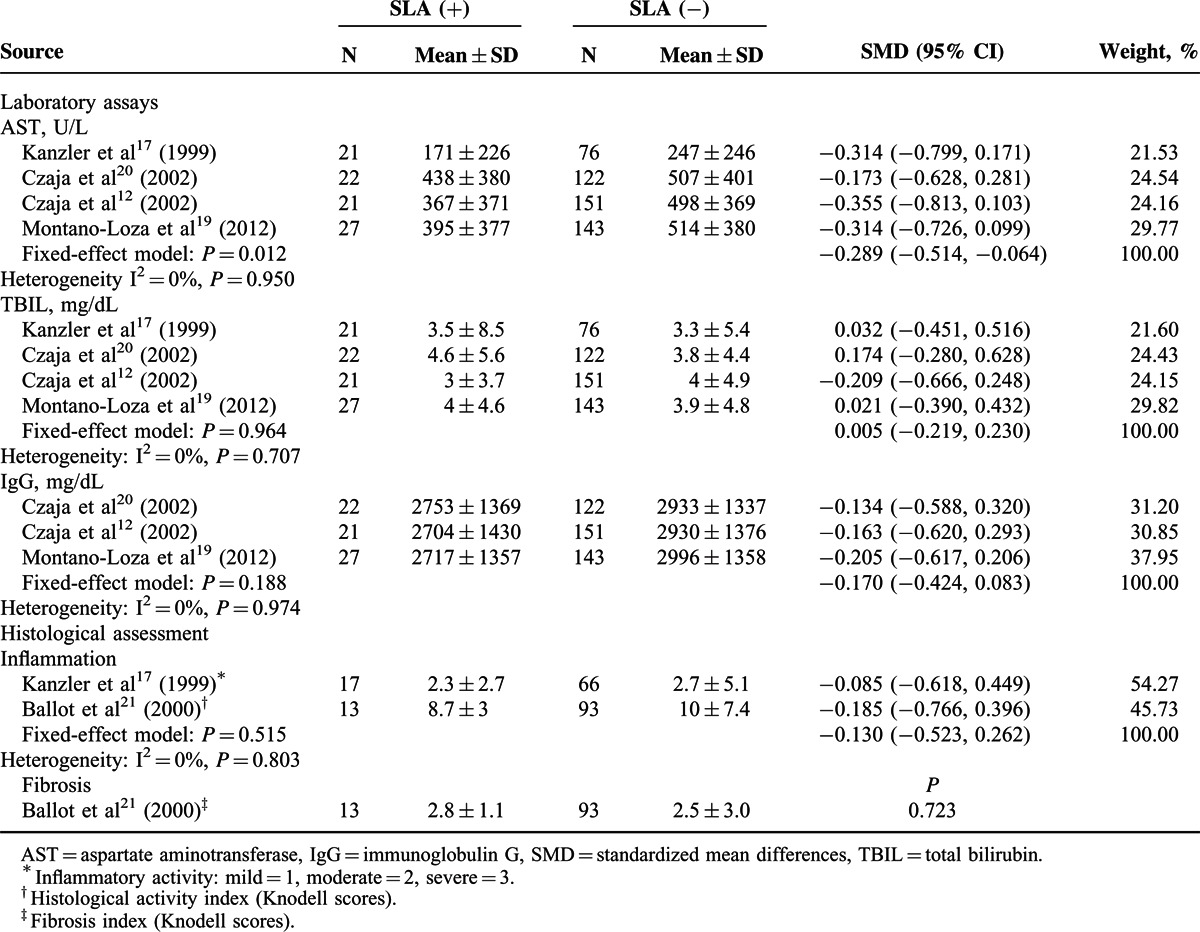
Comparison of Laboratory and Histological Features Between SLA-Positive and SLA-Negative AIH Patients

### Association of Anti-SLA with Disease Prognosis of AIH

Seven studies reported the evaluation of cirrhosis at the diagnosis of AIH.^12–14,17–20^ Among the 8 studies were 3 concerning the frequency of hepatic death,^12,18,20^ 3 concerning the frequency of LT,^12,20,21^ and 4 concerning hepatic death or LT as another outcome measure.^12,14,19,20^

**FIGURE 2:**
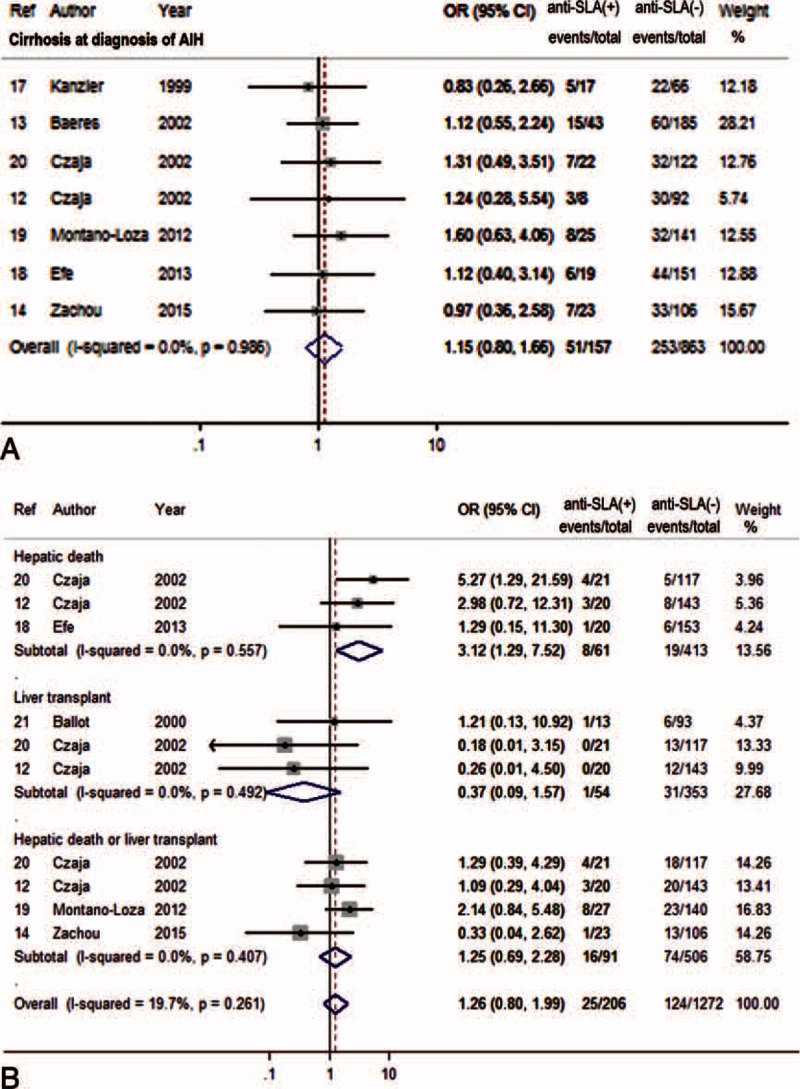
Forest plots of prognostic outcome associations with anti-SLA. The pooled ORs and 95% CIs for risk of cirrhosis at diagnosis (A), or hepatic death and/or liver transplantation (B). CI = confidence interval, OR = odds ratio, SLA = soluble liver antigen.

Figure 2 summarizes characteristics of prognosis of AIH patients with or without anti-SLA. Evaluation of cirrhosis at the diagnosis of AIH revealed that cirrhosis was found in 32% (51/157) of seropositive patients, and in 29% (253/863) of seronegative patients (*P* > 0.05).

Anti-SLA were independently associated with the development of hepatic death (OR = 3.12, 95% CI 1.29–7.52, *P* = 0.011). However, the frequency of LT or the conjunction with hepatic death was comparable between the 2 groups.

### Association of Anti-SLA With Treatment Responses

**FIGURE 3:**
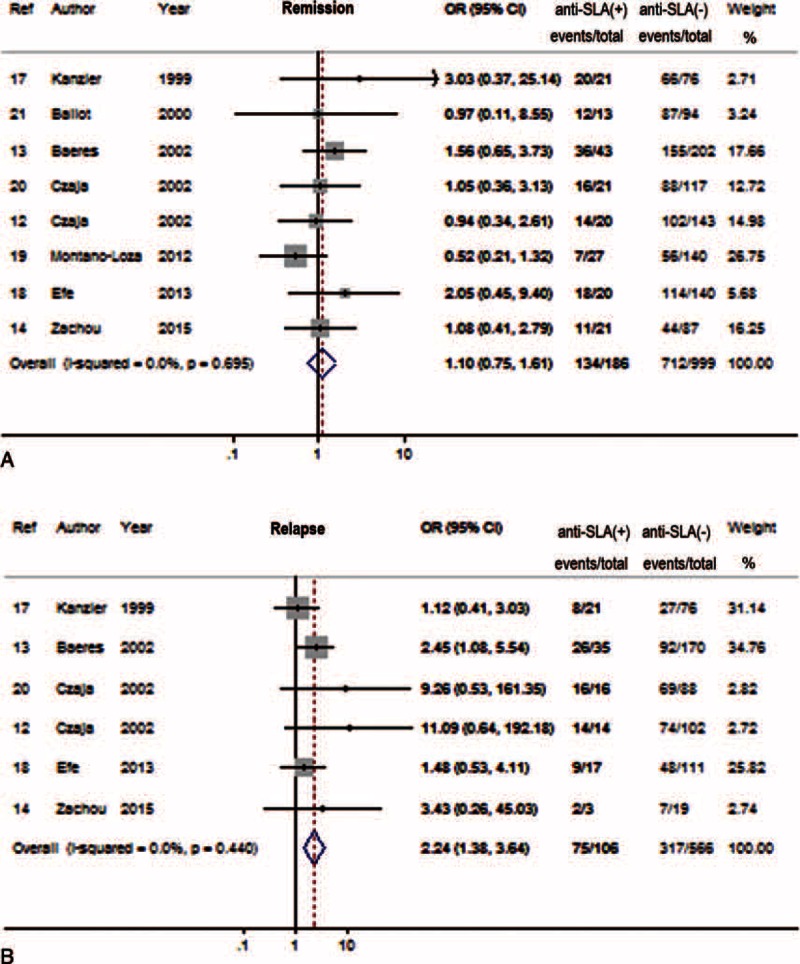
Forest plots of treatment response associations with anti-SLA. The pooled ORs and 95% CIs for remission to immunosuppressive therapy (A), or relapse after drug withdraw (B). CI = confidence interval, OR = odds ratio, SLA = soluble liver antigen.

All the 8 studies reported the remission rates of AIH patients with or without anti-SLA, while only 6 studies covered the relapse. The characteristics of the treatment are described in Table 2. The remission rates were comparable in patients with anti-SLA positive and negative AIH, with 72.0% (134/186) and 71.3 (712/999), respectively (Figure 3A). On the other hand, the meta-analysis showed an association of anti-SLA positivity and risk of relapse after drug (OR 2.24, 95% CI 1.38–3.64, *P* = 0.001) (Figure 3B).

### Association of Anti-SLA With Human Leukocyte Antigen (HLA) DR3 and DR4

**FIGURE 4:**
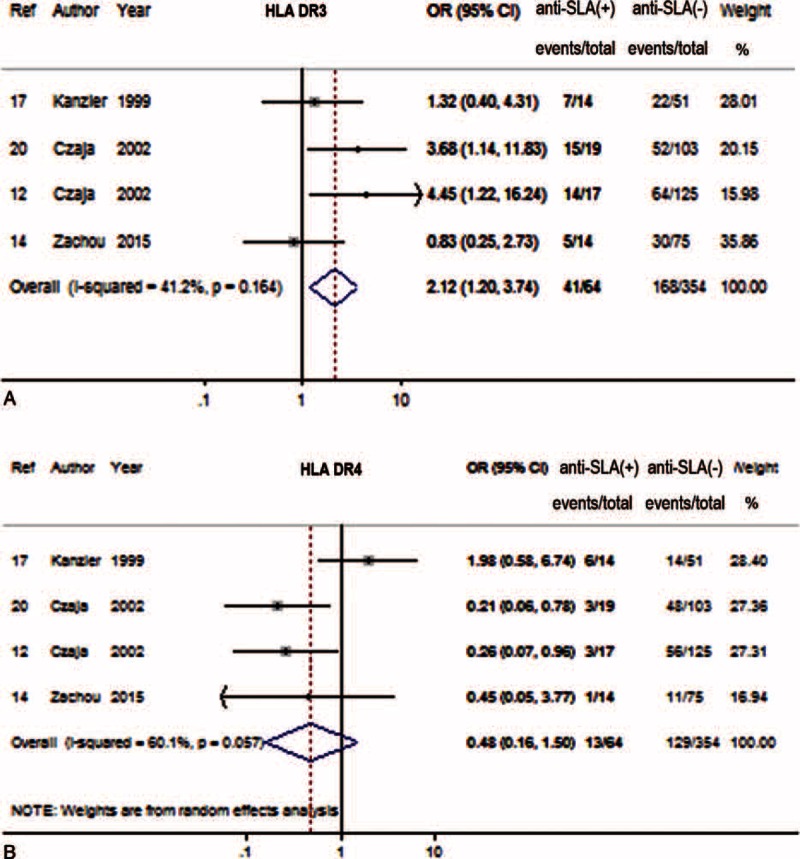
Forest plots of HLA associations with anti-SLA. The pooled ORs and 95% CIs for HLA DR3 (A) and HLA DR4 (B). CI = confidence interval, HLA = human leukocyte antigen allotype, OR = odds ratio, SLA = soluble liver antigen.

For 4 of 8 studies, the HLA status in AIH population was evaluated.^12,14,17,20^ The anti-SLA seropositive patients had HLA allotype DR3 more frequently than seronegative patients (64% vs 47%). Meanwhile, the seropositive patients had HLA DR4 less often than seronegative patients (20% vs 36%). The pooled results indicated that HLA DR3 was positively associated with anti-SLA (OR 2.12, 95% CI 1.20–3.74, *P* = 0.010) (Figure 4A) in the fixed-effect model. Meanwhile, HLA DR4 was not associated with anti-SLA (OR 0.48, 95% CI 0.16–1.50, *P* = 0.019) in the random-effect model (Figure 4B).

### Publication Bias

We assessed publication bias for all pooled ORs with CIs using Egger and Begg tests. The publication bias was *P* < 0.05 in Egger and Begg test, respectively (data not shown). The funnel plots were shown in Figures S1–S3, http://links.lww.com/MD/A294.

## DISCUSSION

There have been very few studies about the prognostic implications of antibodies to SLA, and the conclusions remains controversial.^22^ In the 4 studies included for analysis, serum AST levels tended to be lower in SLA seropositive patients and the difference was statistically significant. The bilirubin and IgG levels were comparable between the 2 groups. Thus, these traditional inflammatory markers might be insensitive to define disease severity here. Moreover, 2 studies with liver biopsy showed that patients with SLA positive or negative AIH had no significant difference in the degree of inflammation or fibrosis.

It was suggested that antibodies to conformational epitopes of SLA defined a severe form of AIH. However, the slightly higher frequency of cirrhosis at diagnosis in patients with anti-SLA did not distinguish them from those without anti-SLA. We also found that the frequency of hepatic death or application of LT, as 1 outcome-measure, was comparable in patients with or without anti-SLA. It is not surprizing because the organ sources are extremely limited and quite a few patients on the waitlist died of liver failure before LT was available. Interestingly, our analysis suggests that the presence of anti-SLA confers an over 3-fold increased risk of hepatic death in patients with AIH.

With regard to the initial response in AIH patients with and without anti-SLA to immunosuppressive therapy, it remains controversial. Kanzler et al^17^ indicated that patients with anti-SLA positive AIH had excellent responses to immunosuppressive therapy and reached complete remission within 1 to 7 months. In contrast, Montano-Loza et al^19^ suggested a less satisfying response. Based on the analysis of data from 8 studies, we found that the overall responses to immunosuppressive therapy did not differ significantly between seropositive and seronegative AIH patients. However, anti-SLA seropositivity was associated with nearly 2-fold increased risk of relapse after drug withdrawal. These finding suggests that the presence of anti-SLA may justify indefinite treatment after the induction of remission.

Concerning the structure of protein, SLA has sequence homology with a short segment of human asialoglycoprotein receptor, which forms part of a hydrophobic membrane-spanning region. Thus, it may be possible that SLA can be inserted into the hepatocyte membrane and be targeted by immunocytes.^20^ Therefore, anti-SLA antibody has a high degree of specificity for AIH and might reflect a pathogenic process more strongly than either antismooth muscle antibody (ASMA) or antinuclear antibody.

Reactivity and titers of autoantibodies may vary during the course of AIH. For instance, ASMA and antinuclear antibody commonly disappear and reappear. Their disappearance was found associated with the improvement of laboratory and histological features of AIH.^23^ A recent study revealed the association between the persistence of high titers of ASMA and/or antiactin antibodies and disease activity in patients with AIH.^24^ With respect to anti-SLA, previous studies indicated that it persisted during the disease and its treatment.^19,20^ However, it remains unknown whether the patients with untreated AIH have this auto-antibody in higher titers and how the titers change during disease progress and treatment course.

HLA DR3 and DR4 are known susceptibility factors in AIH. Czaja et al^25^ suggested that HLA DR3 was associated with early age onset and treatment failure, whereas HLA DR4 was associated with concurrent other immune diseases and remission during immunosuppressive therapy.^26^ Our analysis showed that patients with anti-SLA have HLA DR3 more frequently than patients without. The association of anti-SLA with relapse after drug withdrawal might reflect this allelic association.

Admittedly, our study has some limitations. Our restriction to studies published in English-language means some relevant studies might be missed in particular ethnic groups if they were published in non-English. Fortunately, only a small minority of studies was excluded specifically because they were not in English. Besides, 63% of the enrolled studies were conducted in countries where English is not the primary language. Another limitation of our study is that HCC, which is an important outcome of AIH, has not been covered. Actually, the risk for HCC for patients with liver cirrhosis caused by AIH has not been fully elucidated. Most studies have found that HCC in AIH was relatively uncommon, with the annual incidence of lower than 1.5%,^27–29^ when surveillance in cirrhotic patients is considered to be cost-effective.^30^ Therefore, further studies are needed to determine the association of anti-SLA and the risk of HCC.

In conclusion, our meta-analysis, for the first time, identified that patients with anti-SLA had over 3-fold increased risk of hepatic death and nearly 2-fold increased risk of treatment relapse, compared with seronegative patients. Anti-SLA may serve as a prognostic indicator for AIH. Our findings suggest that this subset of seropositive patients should be maintained indefinitely on individually adjusted medication in order to improve their prognosis.
